# The Two Registration Bills: A Matron's View

**Published:** 1919-04-12

**Authors:** 


### The Two Registration Bills: A Matron's View.

We have received an interesting communication from Miss M. P. Ferrier, "one of the 13,000 nurses already registered," on the subject of the two Registration Bills now before Parliament. We regret it is too long to print in full. Miss Ferrier says:

"The Bill of the Central Committee is not a democratic scheme, the rank and file of the nursing profession taking a secondary place. They claim that 50,000 nurses will register the first year—a truly gigantic number, and one not easily handled in one year unless they have a very large and efficient staff; and they will want premises. If the State decides not to make a grant, where will their funds come from? They appear to be depending for their support on the nurses' registration fees. They are proud and do not want help from outsiders, but the Central Committee forgets that most professions, including the medical profession, have endowments and scholarships.

"The Bill for the College of Nursing, which was not the lucky one in the ballot, is democratic. In this Bill the rank and file are well represented, and during three years they have registered 13,000 nurses. Scholarships have been arranged for with the large hospitals, a bureau has been opened, and has done a great deal towards helping nurses to get appointments both at home and abroad. At present there are local centres in nineteen of our chief towns, to encourage closer co-operation among nurses and provide post-graduate lectures no less than recreation. The College is also working for the improved pay and working hours of the nurse.

"The College has accomplished all this and more in the three years since it first began, for it has funds for endowments, and, knowing that nothing can be done without money it is not too proud to take help from the nation. It must be recognised that more than State Registration is required.

"If the Government does not make a grant for the Bill, the College has funds to the extent of £40,000 already invested, and does not depend on the registration fees of the nurses. Thus it will be able to continue its good work in the interest of the nursing profession."

